# Searching for mechanisms that matter in septic acute kidney injury: an experimental study

**DOI:** 10.1186/cc9517

**Published:** 2011-03-11

**Authors:** J Benes, J Chvojka, R Sykora, J Radej, A Krouzecky, I Novak, M Matejovic

**Affiliations:** 1 Charles University Teaching Hospital, Plzen, Czech Republic; 2 District Hospital, Karlovy Vary, Czech Republic

## Introduction

Both hemodynamic and nonhemodynamic factors are implicated in the pathogenesis of sepsis-induced acute kidney injury (SAKI). However, despite similar septic insult, not all patients develop SAKI. The reasons for the difference in sensitivity to AKI are unknown. Therefore, we sought to analyze dynamic changes in renal hemodynamic and non-hemodynamic responses to sepsis in animals who developed AKI and those who do not.

## Methods

Thirty-six pigs were anesthetized, mechanically ventilated and instrumented. After a recovery period, progressive sepsis was induced either by peritonitis (*n *= 13) or by i.v. infusion of *Pseudomonas aeruginosa *(*n *= 15). Eight sham-operated animals served as time-matched controls. All animals received standard ICU care including goal-directed hemodynamic management. Before and at 12, 18 and 24 hours of sepsis systemic and renal hemodynamic, microcirculatory and inflammatory variables were measured. AKI development was defined using AKIN criteria.

## Results

Fourteen pigs (50%) developed AKI (62% in peritonitis model, 40% in bacteria infusion model) with a significant increase in serum creatinine observed already at 18 hours of sepsis. There were no differences in the systemic hemodynamics and vasopressor support between AKI and non-AKI groups. Although time-dependent reduction in cortical microvascular perfusion was comparable in both groups, only AKI animals developed a progressive increase in renal vascular resistance. This intrarenal vasoconstriction was preceded by a marked overproduction of serum cytokines (TNFα, IL-6) and markers of oxidative stress (TBARS), observed already at 12 hours of sepsis. This induction of proinflammatory response was delayed in non-AKI animals.

## Conclusions

The observed variability in susceptibility to SAKI in our models replicates that of human disease. This heterogeneity allowed us to isolate and study factors discriminating AKI from non-AKI. Early systemic inflammation coupled with late intrarenal vasoconstriction appears to be major determinant of the initiation of SAKI. Genetic and proteomic analyses underlying the observed differences are being analyzed.

